# Racial and Ethnic Differences in the Associations Between COVID-19 Stigma and Mental Health in a Population-Based Study of Adults with SARS-CoV-2 Infection

**DOI:** 10.1089/heq.2023.0241

**Published:** 2024-12-16

**Authors:** Soomin Ryu, Paula Guro, Jana L. Hirschtick, Robert C. Orellana, Nancy L. Fleischer

**Affiliations:** ^1^ Department of Epidemiology, School of Public Health, University of Michigan, Ann Arbor, Michigan, USA.; ^2^ CDC Foundation, Atlanta, Georgia, USA.; ^3^ Michigan Department of Health and Human Services, Lansing, Michigan, USA.

**Keywords:** stigma, COVID-19, mental health, racial disparities, probability sample

## Abstract

**Introduction:**

Many individuals with coronavirus disease 2019 (COVID-19) faced stigmatization, which may contribute to poor health. However, very few studies have explored the relationship between COVID-19 stigma and health, and even less is known about differences in the relationship by race and ethnicity. This article examines associations between COVID-19 stigma and mental health overall and by race and ethnicity.

**Methods:**

We used a population-based probability sample of Michigan adults with SARS-CoV-2 infection between March 2020 and May 2022. We captured COVID-19 stigma based on perceived COVID-19 stigma, fear of COVID-19 disclosure to friends or family, and fear of COVID-19 disclosure at work. We conducted modified Poisson regression with robust standard errors to estimate associations of COVID-19 stigma with depressive and anxiety symptoms adjusting for confounding factors.

**Results:**

Individuals who experienced perceived COVID-19 stigma had 1.44 times higher prevalence of depressive symptoms (95% confidence intervals [CIs]: 1.23–1.69) and 1.48 times higher prevalence of anxiety symptoms (95% CI: 1.30–1.69) compared with individuals who did not experience perceived stigma. Moreover, individuals who were afraid to disclose their COVID-19 diagnosis to friends or family, or who were afraid to disclose their diagnosis at work, had a higher prevalence of depressive symptoms and anxiety symptoms, compared with those who were not afraid. These associations were more pronounced among racial and ethnic minoritized individuals than non-Hispanic White individuals.

**Discussion:**

COVID-19 stigma was associated with depressive and anxiety symptoms. There is a critical need to examine long-lasting effects of stigma, particularly among racial and ethnic minoritized individuals.

## Introduction

The rapid spread of coronavirus disease 2019 (COVID-19) and its associated morbidity and mortality led to disease-related stigma.^1^ Stigma refers to a social process that differentiates people as “abnormal” from the “normal” based on certain characteristics and negative stereotypes.^2–4^ It is common for stigma to be linked with infectious diseases, as stigma has been associated human immunodeficiency virus (HIV), Ebola, and prior sudden acute respiratory syndrome (SARS) epidemics.^5,6^ Fear of new disease and contagion and the spread of misinformation can exacerbate the stigmatization of people infected with diseases.^5,7–11^ The emergence of the COVID-19 pandemic created considerable stress, anxiety, and misinformation about COVID-19.^1,10,12^ Such stress led individuals with suspected or confirmed COVID-19 to experience avoidance, labeling, marginalization, and stigmatization.^13–17^

Stigma is a barrier to seeking health care^5^ and results in heightened stress responses and maladaptive coping behaviors such as smoking and drinking,^5,18–21^ which can ultimately contribute to poor health outcomes. Stigma can also be more detrimental for individuals who have multiple stigmatized social identities simultaneously, such as sex, race and ethnicity, sexual orientation, class, or health conditions.^22–26^ Using the intersectional perspective, some empirical research has studied health disparities among people with disease-related stigma (e.g., HIV) and minoritized social identities (e.g., Black individuals and homosexuality).^23,27,28^

Recent studies have examined the relationship between COVID-19 stigma and mental health outcomes in the United States. Due to a substantial increase in racial discrimination toward people of Asian descent during the COVID-19 pandemic, many studies have focused on Asian Americans, reporting that COVID-19-related stigma was associated with depressive symptoms and anxiety symptoms.^29–32^ Only two population-based studies have examined how COVID-19 stigma was related to mental health outcomes among U.S. adults from diverse racial and ethnic groups. Le and colleagues reported that three measurements of COVID-19-related stigmatization were associated with higher psychological distress and worse self-rated mental health.^33^ Another study also found that COVID-19-related stigmatization was subsequently associated with increased mental distress.^34^ There is a critical need to improve our understanding of the relationship between COVID-19 stigma and mental health among U.S. adults in order to inform public policies to address COVID-19 stigma.

Despite the lack of knowledge about the relationship between COVID-19 stigma and mental health, even less is known about how the relationship is different by race and ethnicity. Growing evidence suggests that racial and ethnic minoritized populations reported higher levels of COVID-19 stigma than non-Hispanic White populations,^34–36^ possibly due to the intersection between their racial and ethnic minority position and disease stigma.^20,23^ In particular, Asian Americans reported a higher level of COVID-19 stigma, racial discrimination, and violence because the stigmatizing terms, such as “Chinese virus” or “Wuhan virus,” were used to refer to COVID-19 by public officials or media as the outbreak of COVID-19 was first reported in Wuhan, China.^32,34,37^ Moreover, it has been well documented that racial and ethnic minoritized populations are also more likely to suffer from a higher prevalence of mental illness compared to non-Hispanic White populations before and during the pandemic.^38–43^ Therefore, it is crucial to examine the differential association of COVID-19 stigma with mental health for minoritized populations.

This article examined the association between COVID-19 stigma and depressive and anxiety symptoms using a population-based probability sample of adults diagnosed with COVID-19 in Michigan. To examine associations at the intersection of COVID-19 stigma and race and ethnicity, we also tested race and ethnicity as potential effect modifiers for the associations between COVID-19 stigma and mental health outcomes.^44^ We hypothesized that individuals who experienced COVID-19 stigma would be more likely to have depressive and anxiety symptoms. We also hypothesized that associations between COVID-19 stigma and mental health outcomes would be more pronounced for racial and ethnic minoritized individuals than non-Hispanic White individuals.

## Methods

### Data

This paper used cross-sectional data from the Michigan COVID-19 Recovery Surveillance Study (MI CReSS), a statewide representative survey of non-institutionalized adults with a polymerase chain reaction (PCR)-confirmed SARS-CoV-2 test, registered in the Michigan Disease Surveillance System (MDSS). A stratified probability sample of adults was selected from geographic strata, including six public health emergency preparedness regions,^45^ six counties in southeast Michigan, and one city (Detroit). We included adults with COVID-19 onset between March 2020 and May 2022. Sixteen sequential cross-sectional samples were drawn over time with a base number of 50 − 70 individuals from each geographic region, while the remainders of the sample were drawn proportionally based on overall case counts within each area. Non-institutionalized adults aged 18 years old or older were eligible for participation in the study if they were alive at the time the survey was conducted and had a valid phone number and zip code or county information. Respondents completed the survey between June 2020 and December 2022 either (1) online in English or (2) over the phone with a trained interviewer in English, Spanish, or Arabic. Survey weights were constructed to match the age and sex distribution of the sampling frame in each geographic area. The median time from COVID-19 illness onset to survey completion was 4.4 months (interquartile range [IQR] = 3.4–5.7 months), and the response rate was 32.1% (American Association for Public Opinion Research response rate #6).^46^

Our analysis excluded respondents with missing information on variables, except for missing household income information, which was imputed using the weighted sequential hot-deck method^47^ and hot deck propensity score imputation.^48^ Of the 5,521 respondents in the 16 MI CReSS samples, 421 were missing outcome or covariate information, including 159 who were missing information on depressive or anxiety symptoms. We also excluded an additional 28 surveys collected via proxy respondents due to mental capacity concerns (*n* = 13) or some other reasons (*n* = 15), such as technological difficulties, time constraint, or physical capacity concerns. We have two separate analytic samples due to the different number of missing records. The full sample for perceived COVID-19 stigma and fear of COVID-19 disclosure to friends or family was *n* = 4,671. The employed sample for fear of COVID-19 disclosure at work was *n* = 3,239, which included only employed respondents. The University of Michigan Institutional Review Board deemed this study exempt due to the use of secondary deidentified data.

### Measures

The outcome variables were depressive symptoms and anxiety symptoms. We measured current depressive symptoms based on responses to the patient health questionnaire 2-item survey, which asks respondents over the last two weeks, “How often have you been bothered by having little interest or pleasure in doing things?” and “How often have you been bothered by feeling down, depressed, or hopeless?”^49^ We assessed current anxiety symptoms using generalized anxiety disorder the 2-item survey, which asks respondents over the last two weeks, “How often have you been bothered by feeling nervous, anxious or on edge?” and “How often have you been bothered by not being able to stop or control worrying?”^50^ Each item was rated on a 4-point Likert scale, with options from 0 (“never”) to 3 (“nearly every day”). We summed ratings within each 2-item construct and created two binary variables to indicate the presence of depressive symptoms (α = 0.87) or anxiety symptoms (α = 0.83) using a cutoff score of 3+, which are widely used, valid, and reliable self-reported psychometric measures.^49,50^

We assessed COVID-19 stigma by using perceived COVID-19 stigma and COVID-19 disclosure stigma (fear of COVID-19 disclosure to friends or family and fear of COVID-19 disclosure at work). Perceived COVID-19 stigma was assessed by using a version of the Everyday Discrimination Scale^51^ that was adapted for use during the COVID-19 pandemic.^52^ We asked whether respondents experienced any of the following situations due to people thinking they might have COVID-19: “you were treated badly/without respect,” “people acted as if they were scared of you,” and “you were threatened or harassed.” We created a binary variable of perceived COVID-19 stigma, which equaled 1 if the respondent answered affirmatively to any of the three items and 0 otherwise. We measured fear of COVID-19 disclosure to friends or family was measured with the question, “Were you afraid or embarrassed to disclose your COVID-19 diagnosis with your friends or family?” Fear of COVID-19 disclosure at work was assessed only among employed respondents using the question, “Were you afraid or embarrassed to disclose your COVID-19 diagnosis at work?” We created binary variables for fear of COVID-19 disclosure to friends or family and fear of COVID-19 disclosure at work (1 = “yes,” 0 = “no”).

We included several sociodemographic and clinical factors as covariates: age-group (18–34, 35–54, 55–64, and ≥65), sex at birth (male and female), race and ethnicity (Hispanic, non-Hispanic White, non-Hispanic Black, and another non-Hispanic race or ethnicity), marital status (married/cohabitating and not currently married/cohabitating), educational attainment (high school or less, some college, and college graduate), household income in 2019 (<$35,000, $35,000–74,999, and ≥$75,000), an indicator for pre-existing diagnosed physical comorbidities (presence of chronic obstructive pulmonary disease, asthma, diabetes, cardiovascular disease, hypertension, liver disease, kidney disease, cerebrovascular disease, cancer, immunosuppressive condition, autoimmune condition, or physical disability), and an indicator for a pre-existing psychological or psychiatric condition. For fear of COVID-19 disclosure at work, a three-level categorical variable for age (18–34, 35–54, and ≥55) was used in the analysis due to the small sample size of employed respondents aged 65 or older.

We used a binary race and ethnicity variable (racial and ethnic minoritized individuals, non-Hispanic White individuals) as a potential effect modifier. Racial and ethnic minoritized individuals included Hispanic, non-Hispanic Black, or another non-Hispanic race or ethnicity. We were not able to use disaggregated race and ethnicity due to small sample sizes of some of the racial and ethnic subgroups.

### Statistical analysis

First, we calculated descriptive statistics for the two analytic samples. Next, we calculated weighted prevalence estimates of the three measures of COVID-19 stigma overall and by sociodemographic and health status covariates. Then, we conducted unadjusted and adjusted modified Poisson regression models with robust standard errors to estimate prevalence ratios (PR) for the association of each COVID-19 stigma measure with depressive or anxiety symptoms, separately. Adjusted regression models included all covariates, survey mode (phone or online), and pandemic phase. Additionally, we examined the association between each item of perceived COVID-19 stigma and depressive or anxiety symptoms. Finally, we tested race and ethnicity as potential effect modifiers for the associations between COVID-19 stigma and mental health and examined associations stratified by race and ethnicity. We conducted an additional analysis using non-Hispanic White adults not experiencing stigma as a referent group for three other comparative groups: racial and ethnic minoritized adults not experiencing stigma, non-Hispanic White adults experiencing stigma, and racial and ethnic minoritized adults experiencing stigma. All statistical analyses were completed using Stata, version 17, and incorporated sampling strata and survey weights.

## Results

Perceived COVID-19 stigma (35.4%), fear of COVID-19 disclosure to friends or family (20.7%), and fear of COVID-19 disclosure at work (15.6%) were prevalent among adults with COVID-19 (Table 1). Depressive symptoms were reported by 13–14% of respondents and more than 17% reported anxiety symptoms. All three measures of COVID-19 stigma were more prevalent among individuals who had depressive symptoms (48.3% for perceived stigma, 31.6% for disclosure stigma to friends/family, and 29.3% for disclosure stigma at work) than among individuals who did not have depressive symptoms (31.0%, 19.0%, 13.6%) and were also more prevalent among individuals who had anxiety symptoms (48.9%, 32.9%, 30.6%) than among individuals who did not have anxiety symptoms (30.0%, 18.1%, 12.5%; Table 2). By race and ethnicity, perceived COVID-19 was most prevalent among individuals who were non-Hispanic Black (40.8%), while fear of COVID-19 disclosure was most prevalent among Hispanic individuals (27.9% to friends/family, 20.1% at work). All three measures of COVID-19 stigma were the least prevalent among individuals who were non-Hispanic White (31.6% for perceived stigma, 18.9% for disclosure stigma to friends/family, and 14.8% for disclosure stigma at work).

**Table 1.** Characteristics of Study Respondents, Michigan COVID-19 Recovery Surveillance Study, 2020–2022

	Full sample^a^ (*n* = 4,671)	Employed sample^b^ (*n* = 3,239)
	Weighted percentage	Weighted percentage
Perceived COVID-19 stigma		
Yes	35.4	
Fear of COVID-19 disclosure to friends or family		
Yes	20.7	
Fear of COVID-19 disclosure at work		
Yes		15.6
Depressive symptoms		
Yes	14.0	13.1
Anxiety symptoms		
Yes	17.9	17.5
Age		
18–34	37.1	
35–54	35.0	
55–64	15.6	
≥65	12.3	
Age^c^		
18–34		38.6
35–54		41.3
≥55		20.1
Sex		
Male	45.0	46.8
Female	55.0	53.2
Race and ethnicity		
Hispanic	7.4	7.9
Non-Hispanic White	71.9	72.7
Non-Hispanic Black	9.4	8.6
Another non-Hispanic race and ethnicity	11.3	10.8
Marital status		
Married/cohabiting	62.3	65.0
Not married/cohabiting	37.7	35.0
Education		
Less than high school	24.2	21.5
Some college	34.2	32.4
College graduate	41.7	46.1
Household income		
<$35,000	30.0	25.1
$35,000–74,999	30.6	31.3
≥$75,000	39.5	43.7
Pre-existing diagnosed physical comorbidities		
Yes	49.4	43.8
Pre-existing diagnosed psychological/psychiatric condition		
Yes	13.8	12.5

^a^ Full sample was for perceived COVID-19 stigma and fear of COVID-19 disclosure to friends or family.

^b^ Employed sample was for fear of COVID-19 disclosure at work, which included only employed respondents.

^c^ Three-level categorical variable of age (18–34, 35–54, and ≥55 years) was used in the analysis for fear of COVID-19 disclosure at work due to small sample size of employed respondents aged 65 years or older.

COVID-19, coronavirus disease 2019.

**Table 2.** Weighted Prevalence of COVID-19 Stigma Measures by Sociodemographic and Clinical Factors, Michigan COVID-19 Recovery Surveillance Study, 2020–2022

	Perceived COVID-19 stigma (*n* = 4,671)		Fear of COVID-19 disclosure to friends/family (*n* = 4,671)		Fear of COVID-19 disclosure at work (*n* = 3,239)	
	Weighted row (%)	95% CI (LB, UB)	Weighted row (%)	95% CI (LB, UB)	Weighted row (%)	95% CI (LB, UB)
Depressive symptoms						
Yes	48.3	(44.1, 52.6)	31.6	(27.8, 35.7)	29.3	(24.8, 34.3)
No	31.0	(29.4, 32.6)	19.0	(17.7, 20.3)	13.6	(12.3, 15.0)
Anxiety symptoms						
Yes	48.9	(45.2, 52.7)	32.9	(29.5, 36.5)	30.6	(26.6, 34.9)
No	30.0	(28.4, 31.6)	18.1	(16.8, 19.5)	12.5	(11.2, 13.9)
Age						
18–34	37.2	(34.6, 39.9)	28.2	(25.8, 30.7)		
35–54	35.3	(32.8, 37.9)	20.3	(18.3, 22.5)		
55–64	29.4	(26.0, 33.0)	13.2	(10.9, 15.9)		
≥65	21.5	(18.4, 25.0)	9.1	(7.0, 11.7)		
Age^a^						
18–34					19.9	(17.5, 22.5)
35–54					14.6	(12.7, 16.8)
≥55					9.6	(7.5, 12.1)
Sex						
Male	28.4	(26.2, 30.7)	13.6	(12.2, 15.5)	11.1	(9.4, 13.1)
Female	37.5	(35.5, 39.5)	26.5	(24.8, 28.4)	19.6	(17.7, 21.7)
Race and ethnicity						
Hispanic	39.0	(33.3, 45.1)	27.9	(22.8, 33.6)	20.1	(15.0, 26.3)
Non-Hispanic White	31.6	(29.9, 33.3)	18.9	(17.5, 20.3)	14.8	(13.3, 16.4)
Non-Hispanic Black	40.8	(35.9, 45.9)	26.1	(21.8, 30.8)	17.4	(13.1, 22.8)
Another non-Hispanic race/ethnicity	35.2	(30.7, 40.0)	23.7	(19.8, 28.0)	16.5	(12.7, 21.3)
Marital status						
Married/cohabiting	31.6	(29.8, 33.5)	19.3	(17.8, 21.0)	14.6	(13.2, 16.3)
Not married/cohabiting	36.4	(33.9, 38.9)	23.2	(21.0, 25.5)	17.7	(15.3, 20.2)
Education						
Less than high school	34.8	(31.8, 37.9)	19.6	(17.2, 22.3)	14.0	(11.4, 17.2)
Some college	35.2	(32.7, 37.9)	19.7	(17.6, 21.9)	14.8	(12.6, 17.3)
College graduate	31.1	(28.9, 33.4)	22.3	(20.3, 24.4)	17.0	(15.0, 19.2)
Household income						
<$35,000	37.7	(35.0, 40.6)	23.1	(20.8, 25.6)	18.0	(15.3, 21.1)
$35,000–74,999	34.6	(31.9, 37.3)	21.1	(18.8, 23.6)	16.9	(14.5, 19.6)
≥$75,000	29.2	(26.9, 31.5)	18.7	(16.8, 20.7)	13.4	(11.6, 15.4)
Pre-existing diagnosed physical comorbidities						
Yes	34.3	(32.3, 36.5)	18.4	(16.8, 20.2)	15.8	(13.9, 17.9)
No	32.5	(30.4, 34.6)	23.0	(21.2, 25.0)	15.5	(13.8, 17.4)
Pre-existing diagnosed psychological/psychiatric condition						
Yes	45.8	(41.6, 50.0)	31.6	(27.8, 35.6)	28.7	(24.1, 33.7)
No	31.4	(29.8, 33.0)	19.0	(17.7, 20.4)	13.8	(12.5, 15.2)

Weighted percentages are reported.

^a^ Three-level categorical variable of age (18–34, 35–54, and ≥55 years) was used in the analysis for fear of COVID-19 disclosure at work due to small sample size of employed respondents aged 65 years or older.

CI, confidence interval; COVID-19, coronavirus disease 2019; LB, lower bound; UB, upper bound.

Table 3 reports the results of Poisson regression models examining associations between COVID-19 stigma and depressive and anxiety symptoms. In the unadjusted model, individuals with perceived COVID-19 stigma had 1.86 times higher prevalence of reporting depressive symptoms (95% confidence intervals [CIs]: 1.59–2.18) and 1.91 times higher prevalence of reporting anxiety symptoms (95% CI: 1.67–2.19) compared with individuals without perceived COVID-19 stigma. After covariate adjustment, the magnitude of these associations was attenuated to 1.44 (95% CI: 1.23–1.69) and 1.48 (95% CI: 1.30–1.69), although they remained statistically significant. Additionally, in the unadjusted models, individuals who were afraid of disclosing their COVID-19 diagnosis to friends or family had 1.77 times (95% CI: 1.50–2.09) higher prevalence of reporting depressive symptoms and 1.87 times (95% CI: 1.63–2.15) higher prevalence of reporting anxiety symptoms compared to individuals who were not afraid to disclose their diagnosis to friends or family. With adjustment of covariates, the magnitude of the associations was slightly attenuated (adjusted PR [aPR]: 1.39, 95% CI: 1.17–1.64 for depressive symptoms; aPR: 1.41; 95% CI: 1.23–1.62 for anxiety symptoms). In adjusted models, employed individuals who were afraid to disclose COVID-19 diagnosis at work were also more likely to report depressive symptoms (aPR: 1.65, 95% CI: 1.34–2.04) and anxiety symptoms (aPR: 1.71, 95% CI: 1.44–2.04) compared with employed individuals who were not afraid to disclose their diagnosis at work. In an additional analysis, we found that each item of perceived COVID-19 stigma was associated with a higher prevalence of reporting depressive symptoms and anxiety symptoms (Supplementary Table S1).

**Table 3.** Poisson Regression: Association of COVID-19 Stigma Measures with Depressive and Anxiety Symptoms, Michigan COVID-19 Recovery Surveillance Study, 2020–2022

	Depressive symptoms (yes/no) PR (95% CI)		Anxiety symptoms (yes/no) PR (95% CI)	
	Unadjusted model	Adjusted model	Unadjusted model	Adjusted model
Perceived COVID-19 stigma	1.86 (1.59–2.18)	1.44 (1.23–1.69)	1.91 (1.67–2.19)	1.48 (1.30–1.69)
Fear of COVID-19 disclosure to friends or family	1.77 (1.50–2.09)	1.39 (1.17–1.64)	1.87 (1.63–2.15)	1.41 (1.23–1.62)
Fear of COVID-19 disclosure at work	2.23 (1.82–2.74)	1.65 (1.34–2.04)	2.38 (2.02–2.80)	1.71 (1.44–2.04)

Adjusted models included age, sex at birth, race and ethnicity, marital status, education, household income, pre-existing diagnosed physical comorbidities, pre-existing diagnosed psychological/psychiatric condition, survey mode, and pandemic phase.

CI, confidence interval; COVID-19, coronavirus disease 2019; PR, prevalence ratio.

We tested race and ethnicity as potential effect modifiers of the associations between COVID-19 stigma and mental health outcomes. Although race and ethnicity were not statistically significant effect modifiers at *p* < 0.05, the stratified analysis results suggest that associations between COVID-19 stigma and mental health outcomes were more pronounced among racial and ethnic minoritized individuals compared to non-Hispanic White individuals (Table 4). To further explore the relationship at the intersection of COVID-19 stigma and race and ethnicity, we examined associations using non-Hispanic White individuals not experiencing stigma as the referent group (Supplementary Table S2). Compared with non-Hispanic White individuals without COVID-19 stigma, individuals with COVID-19 stigma had higher prevalence of depressive and anxiety symptoms, and these associations became even stronger for racial and ethnic minoritized individuals in terms of both magnitude and statistical significance.

**Table 4.** Stratified by Race and Ethnicity: Association of COVID-19 Stigma Measures with Depressive and Anxiety Symptoms, Michigan COVID-19 Recovery Surveillance Study, 2020–2022

	Depressive symptoms (yes/no) aPR (95% CI)		Anxiety symptoms (yes/no) aPR (95% CI)	
	Racial/ethnic minoritized individuals	Non-Hispanic White individuals	Racial/ethnic minoritized individuals	Non-Hispanic White individuals
Perceived COVID-19 stigma	1.63 (1.25–2.11)	1.36 (1.12–1.66)	1.60 (1.27–2.02)	1.41 (1.20–1.65)
*p*-Value of stigma × race/ethnicity	0.708		0.547	
Fear of COVID-19 disclosure to friends or family	1.64 (1.26–2.14)	1.31 (1.05–1.62)	1.73 (1.38–2.18)	1.30 (1.09–1.54)
*p*-Value of stigma × race/ethnicity	0.926		0.532	
Fear of COVID-19 disclosure at work	1.73 (1.22–2.45)	1.58 (1.21–2.05)	1.94 (1.45–2.58)	1.67 (1.34–2.07)
*p*-Value of stigma × race/ethnicity	0.173		0.619	

Covariates are age, sex at birth, marital status, education, household income, pre-existing diagnosed physical comorbidities, pre-existing diagnosed psychological/psychiatric condition, survey mode, and pandemic phase. Racial and ethnic minoritized individuals included Hispanic, non-Hispanic Black, and another non-Hispanic race or ethnicity.

aPR, adjusted prevalence ratio; CI, confidence interval; COVID-19, coronavirus disease 2019.

## Discussion

We examined the association between COVID-19 stigma and mental health outcomes using a population-based sample of adults who tested positive for SARS-CoV-2 in Michigan between 2020 and 2022. We found that perceived COVID-19 stigma and COVID-19 disclosure stigma were associated with depressive and anxiety symptoms, supporting our first hypothesis. Our results are consistent with previous findings of an association between COVID-19-related discrimination or stigma and poor mental health among U.S. adults.^33,34,53^ Interestingly, fear of disclosing COVID-19 at work was the least prevalent among the stigma measurements, but it had the strongest association with depressive symptoms and anxiety symptoms compared to perceived COVID-19 stigma and fear of disclosing COVID-19 to friends or family. One potential explanation for this finding may be related to workplace conditions.^54,55^ For example, people who are able to use paid sick leave or work remotely may not be afraid of disclosing their COVID-19 diagnosis at work due to workplace accommodations supportive of recovery, while people without sick leave or remote work options may not want to disclose their illness at work so that they can continue to work.^55^ Additionally, other research documented that individuals believe that co-workers are less likely to provide continuous support after disclosure of their illness compared to friends or family.^56,57^

We also examined the effect modification of COVID-19 stigma and mental health outcomes by race and ethnicity. Although the statistical interactions between stigma and race and ethnicity were not statistically significant, we found that individuals who were from racial and ethnic minoritized backgrounds had more pronounced associations between COVID-19 stigma and depressive or anxiety symptoms than those who were non-Hispanic Whites. Furthermore, in the additional analysis, we found that racial and ethnic minoritized individuals experiencing stigma had the strongest associations with depressive and anxiety symptoms when compared to non-Hispanic White individuals who did not experience stigma. Thus, the results support our second hypothesis that the associations would be more pronounced for racial and ethnic minoritized individuals compared to non-Hispanic White individuals. Our finding is aligned with prior studies that suggested stigma was worse among people who had minoritized social identities as well as illness compared to people who had only illness.^22,23^ According to these studies, individuals who have diseases like HIV/acquired immunodeficiency syndrome (AIDS) and were African American, female, or homosexual experienced compounding effects of their illness stigma and their social identity stigma.^23^ Our results on the intersection of COVID-19 stigma and race and ethnicity highlight that racial and ethnic minoritized populations with confirmed COVID-19 might also experience compounding effects of their illness stigma and stigma from minoritized identity during the pandemic.

This study has some limitations to highlight. First, the finding may have limited generalizability as our sample includes only individuals who received a positive PCR test for SARS-CoV-2 and were recorded in the MDSS with valid contact and geographic information and who were alive when the survey was conducted. Nonetheless, this is among the few population-based studies that incorporate a probability sampling approach to examine the impact of COVID-19 illness on health. Second, we assessed mental health using self-reported depressive and anxiety symptoms that do not indicate clinical diagnoses. Third, due to cross-sectional data, we were not able to observe casual mechanisms. Fourth, we were not able to examine effect modification of the associations between COVID-19 stigma and mental health using more disaggregated racial and ethnic categories due to small sample sizes of some of the racial and ethnic subgroups. In particular, given that people of Asian descent experienced severe racial discrimination during the early COVID-19 pandemic,^29–32^ the differential association among Asian Americans is an important consideration for future work with larger Asian subsamples. Finally, we were not able to examine whether personal or social resources, such as self-esteem and social support,^58–62^ buffer the relationship between COVID-19 stigma and mental health due to the lack of information in our survey tool.

### Public Health Implications

Reducing COVID-19 stigma has implications not only for people with COVID-19 but also for the general population and society because disclosing COVID-19 illness is necessary to mitigate the spread of COVID-19.^56,63^ Therefore, governments should consider providing anti-stigma campaigns to address misconceptions about COVID-19 and reduce negative consequences of stigma on health. Continuing to monitor the long-term effects of COVID-19 stigma on mental health, particularly among racial and ethnic minoritized populations, will provide useful insight for future pandemics.

## Ethical Approval

Ethical approval for this analysis was considered exempt by the University of Michigan Institutional Review Board due to the use of a deidentified secondary dataset. Informed consent was obtained from all individual respondents included in this study.

## Data Availability

Although the dataset used in this study is not currently available to others, we are in the process of making a deidentified dataset and data dictionary publicly available.

## Abbreviations Used

CI: confidence interval
COVID-19: coronavirus disease 2019
IQR: interquartile range
MDSS: Michigan Disease Surveillance System
MI CReSS: Michigan COVID-19 Recovery Surveillance Study
PCR: polymerase chain reaction
PR: prevalence ratios
SARS: sudden acute respiratory syndrome
