# Supplementary material for: Racial and Ethnic Differences in the Associations Between COVID-19 Stigma and Mental Health in a Population-Based Study of Adults with SARS-CoV-2 Infection
Source: Health Equity. 2024 Dec 16;8(1):790–9. doi: 10.1089/heq.2023.0241 (PMC11671311; doi:10.1089/heq.2023.0241)
Supplement: Supplementary Table S1 [file heq.2023.0241_supp_tables1.docx]

**Supplementary Tables**

**Racial and ethnic differences in the associations between COVID-19 stigma and mental health in a population-based study of adults with SARS-CoV-2 infection**

Soomin Ryu, Paula Guro, Jana L. Hirschtick, Robert C. Orellana, and Nancy L. Fleischer

**Supplemental Table S1.** Poisson regression: Association of each item of perceived COVID-19 stigma with depressive and anxiety symptoms, Michigan COVID-19 Recovery Surveillance Study, 2020-2022

|  | **Depressive symptoms (yes/no)** | | **Anxiety symptoms (yes/no)** | |
| --- | --- | --- | --- | --- |
|  | **PR (95% CI)** | | **PR (95% CI)** | |
|  | Unadjusted model | Adjusted model | Unadjusted model | Adjusted model |
| Being treated badly/without respect | 2.15 (1.80-2.56) | 1.55 (1.29-1.85) | 2.04 (1.76-2.38) | 1.48 (1.27-1.73) |
|  |  |  |  |  |
| Scared of you | 1.85 (1.58-2.16) | 1.46 (1.25-1.71) | 1.95 (1.70-2.22) | 1.54 (1.35-1.76) |
|  |  |  |  |  |
| Being threatened/harassed | 2.23 (1.69-2.95) | 1.51 (1.15-2.00) | 1.86 (1.44-2.42) | 1.32 (1.02-1.71) |

Note: Adjusted models included age, sex at birth, race and ethnicity, marital status, education, household income, pre-existing diagnosed physical comorbidities, pre-existing diagnosed psychological/psychiatric condition, survey mode, and pandemic phase.

COVID-19=coronavirus disease 2019, PR=prevalence ratio, CI=confidence interval.
