# Supplementary material for: Racial and Ethnic Differences in the Associations Between COVID-19 Stigma and Mental Health in a Population-Based Study of Adults with SARS-CoV-2 Infection
Source: Health Equity. 2024 Dec 16;8(1):790–9. doi: 10.1089/heq.2023.0241 (PMC11671311; doi:10.1089/heq.2023.0241)
Supplement: Supplementary Table S2 [file heq.2023.0241_supp_tables2.docx]

**Supplemental Table S2.** Poisson regression for depressive and anxiety symptoms stratified by COVID-19 stigma measures and race and ethnicity, Michigan COVID-19 Recovery Surveillance Study, 2020-2022

|  | **Depressive symptoms (yes/no)** | | **Anxiety symptoms**  **(yes/no)** | |
| --- | --- | --- | --- | --- |
|  | aPR (95% CI) | P-value | aPR (95% CI) | P-value |
| **Perceived COVID-19 stigma** |  |  |  |  |
| Non-Hispanic White, no stigma | 1.0 (ref) |  | 1.0 (ref) |  |
| Racial/ethnic minoritized, no stigma | 1.14 (0.89-1.47) | 0.306 | 0.98 (0.79-1.22) | 0.841 |
| Non-Hispanic White, with stigma | 1.36 (1.12-1.66) | 0.002 | 1.41 (1.20-1.65) | <0.001 |
| Racial/ethnic minoritized, with stigma | 1.60 (1.25-2.05) | <0.001 | 1.50 (1.22-1.84) | <0.001 |
|  |  |  |  |  |
| **Fear of COVID-19 disclosure to friends or family** |  |  |  |  |
| Non-Hispanic White, no stigma | 1.0 (ref) |  | 1.0 (ref) |  |
| Racial/ethnic minoritized, no stigma | 1.10 (0.89-1.36) | 0.381 | 0.94 (0.78-1.13) | 0.504 |
| Non-Hispanic White, with stigma | 1.31 (1.05-1.62) | 0.014 | 1.30 (1.09-1.56) | 0.004 |
| Racial/ethnic minoritized, with stigma | 1.60 (1.24-2.07) | <0.001 | 1.45 (1.18-1.78) | <0.001 |
|  |  |  |  |  |
| **Fear of COVID-19 disclosure at work** |  |  |  |  |
| Non-Hispanic White, no stigma | 1.0 (ref) |  | 1.0 (ref) |  |
| Racial/ethnic minoritized, no stigma | 1.22 (0.94-1.59) | 0.140 | 1.04 (0.83-1.29) | 0.760 |
| Non-Hispanic White, with stigma | 1.58 (1.21-2.05) | 0.001 | 1.66 (1.34-2.06) | <0.001 |
| Racial/ethnic minoritized, with stigma | 1.94 (1.37-2.74) | <0.001 | 1.74 (1.34-2.28) | <0.001 |

Note: Covariates are age, sex at birth, marital status, education, household income, pre-existing diagnosed physical comorbidities, pre-existing diagnosed psychological/psychiatric condition, survey mode and pandemic phase. Racial and ethnic minoritized individuals included Hispanic, non-Hispanic Black, or another non-Hispanic race or ethnicity.

COVID-19 =coronavirus disease 2019, aPR=adjusted prevalence ratio, CI=confidence interval, ref=reference.
